# Laparoscopic uterine-preserving management of ruptured pyometra using open drainage: a case report

**DOI:** 10.1093/jscr/rjag076

**Published:** 2026-02-17

**Authors:** Yuki Hirano, Hiroshi Sato, Yusuke Yamaoka, Masumi Kiyose, Yumiko Takeuchi, Fumika Hamaguchi, Seigo Ibuchi, Yuri Takemura, Yumi Onishi, Yu Takaishi, Satomi Kan, Miho Masuda, Kanako Kawaharamura, Yukiko Ando, Nao Taguchi, Kazuyo Kakui

**Affiliations:** Department of Obstetrics and Gynecology, Hyogo Prefectural Amagasaki General Medical Center, 2-17-77 Higashinaniwa Amagasaki Hyogo, Japan; Department of Obstetrics and Gynecology, Hyogo Prefectural Amagasaki General Medical Center, 2-17-77 Higashinaniwa Amagasaki Hyogo, Japan; Department of Obstetrics and Gynecology, Hyogo Prefectural Amagasaki General Medical Center, 2-17-77 Higashinaniwa Amagasaki Hyogo, Japan; Department of Obstetrics and Gynecology, Hyogo Prefectural Amagasaki General Medical Center, 2-17-77 Higashinaniwa Amagasaki Hyogo, Japan; Department of Obstetrics and Gynecology, Hyogo Prefectural Amagasaki General Medical Center, 2-17-77 Higashinaniwa Amagasaki Hyogo, Japan; Department of Obstetrics and Gynecology, Hyogo Prefectural Amagasaki General Medical Center, 2-17-77 Higashinaniwa Amagasaki Hyogo, Japan; Department of Obstetrics and Gynecology, Hyogo Prefectural Amagasaki General Medical Center, 2-17-77 Higashinaniwa Amagasaki Hyogo, Japan; Department of Obstetrics and Gynecology, Hyogo Prefectural Amagasaki General Medical Center, 2-17-77 Higashinaniwa Amagasaki Hyogo, Japan; Department of Obstetrics and Gynecology, Hyogo Prefectural Amagasaki General Medical Center, 2-17-77 Higashinaniwa Amagasaki Hyogo, Japan; Department of Obstetrics and Gynecology, Hyogo Prefectural Amagasaki General Medical Center, 2-17-77 Higashinaniwa Amagasaki Hyogo, Japan; Department of Obstetrics and Gynecology, Hyogo Prefectural Amagasaki General Medical Center, 2-17-77 Higashinaniwa Amagasaki Hyogo, Japan; Department of Obstetrics and Gynecology, Hyogo Prefectural Amagasaki General Medical Center, 2-17-77 Higashinaniwa Amagasaki Hyogo, Japan; Department of Obstetrics and Gynecology, Hyogo Prefectural Amagasaki General Medical Center, 2-17-77 Higashinaniwa Amagasaki Hyogo, Japan; Department of Obstetrics and Gynecology, Hyogo Prefectural Amagasaki General Medical Center, 2-17-77 Higashinaniwa Amagasaki Hyogo, Japan; Department of Obstetrics and Gynecology, Hyogo Prefectural Amagasaki General Medical Center, 2-17-77 Higashinaniwa Amagasaki Hyogo, Japan; Department of Obstetrics and Gynecology, Hyogo Prefectural Amagasaki General Medical Center, 2-17-77 Higashinaniwa Amagasaki Hyogo, Japan

**Keywords:** ruptured pyometra, laparoscopic surgery, uterine-preserving surgery, open drainage, generalized peritonitis, source control

## Abstract

Ruptured pyometra is a rare but life-threatening condition that often presents with generalized peritonitis and septic shock, particularly in elderly patients. Although total hysterectomy with peritoneal lavage has traditionally been the standard treatment, it may impose excessive physiological stress in patients with poor general condition. We report a case of an elderly woman with ruptured pyometra who was treated with laparoscopic uterine-preserving management using open drainage. Intraoperatively, uterine perforations with purulent discharge were identified. Instead of closing the perforation, the uterine wall was intentionally incised to widely open the abscess cavity, allowing thorough irrigation of the uterine cavity and sustained drainage. This approach prioritized effective source control over anatomical repair. The postoperative course was favorable, and recurrent infection was successfully managed with additional minimally invasive drainage. This case suggests that laparoscopic open drainage without closure of uterine perforation may be a viable, life-saving option in selected high-risk patients.

## Introduction

Spontaneous rupture of pyometra is extremely rare (0.01%–0.05%) but potentially fatal, often leading to generalized peritonitis or septic shock with a reported mortality of 15%–20% [1, 2]. Open hysterectomy with peritoneal lavage has traditionally been considered the standard treatment [3, 4]. However, this approach may impose excessive physiological stress in elderly patients with poor general condition. We report a case of ruptured pyometra in an 88-year-old woman who was successfully treated with laparoscopic uterine-preserving management using open drainage.

## Case presentation

An 88-year-old multiparous woman with declining activities of daily living (Performance Status 3) presented with general fatigue and vomiting. Abdominal computed tomography (CT) at the referring hospital showed free intraperitoneal gas, and she was transferred with suspected gastrointestinal perforation. Her medical history included diabetes mellitus, prior myocardial infarction, chronic heart failure, and chronic kidney disease.

**Figure 1:**
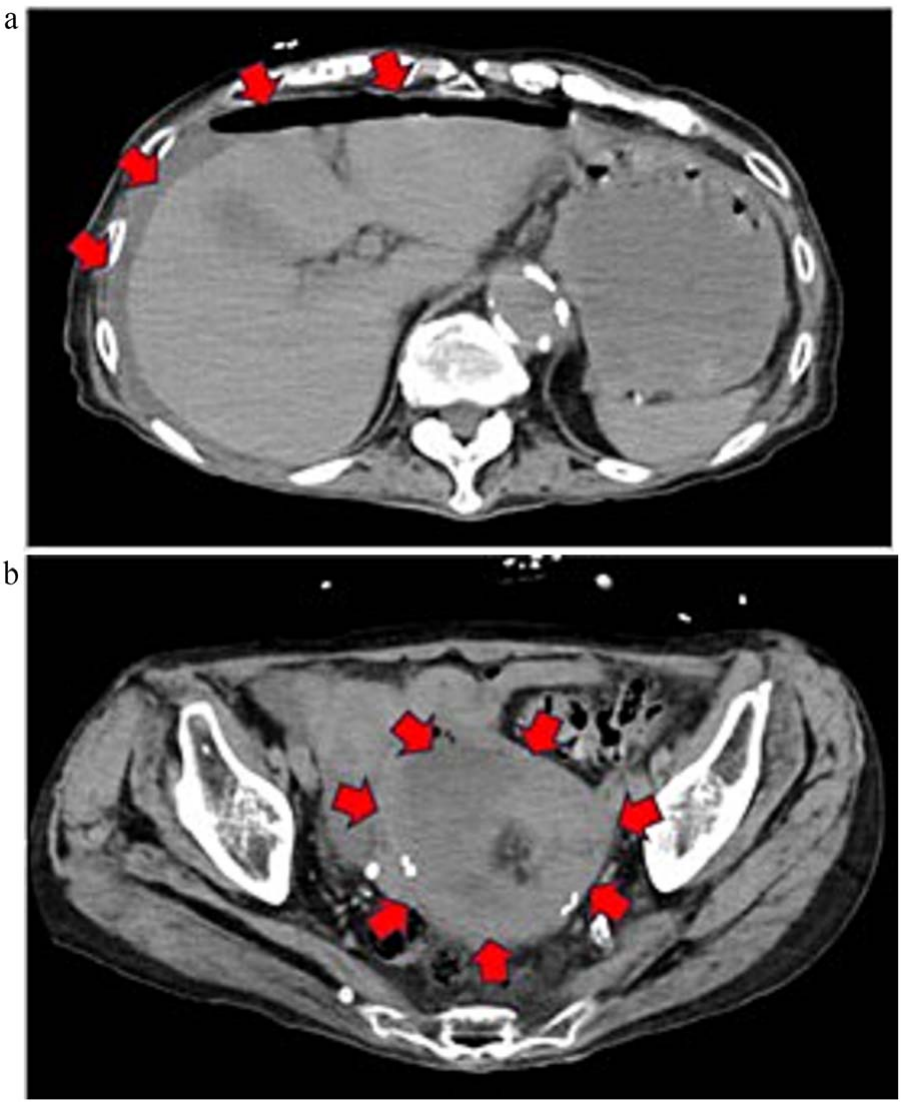
Preoperative contrast-enhanced CT image (equilibrium phase). (a) Horizontal section. Extensive ascites extending to the liver surface and free gas in the abdominal cavity are noted. (b) Horizontal section. A low-attenuation area suggestive of a retained uterine abscess was identified within the uterus.

On admission, she was in shock (blood pressure 73/52 mmHg, pulse 113 bpm) with diffuse abdominal tenderness. Laboratory tests revealed marked inflammation (WBC 10.7 × 10^3^/μL, CRP 12.49 mg/dL) and hypoalbuminemia (2.2 g/dL). CT demonstrated massive ascites with free gas and a low-density uterine lesion suggestive of pyometra (Fig. 1).

### Surgical findings and procedure

**Figure 2:**
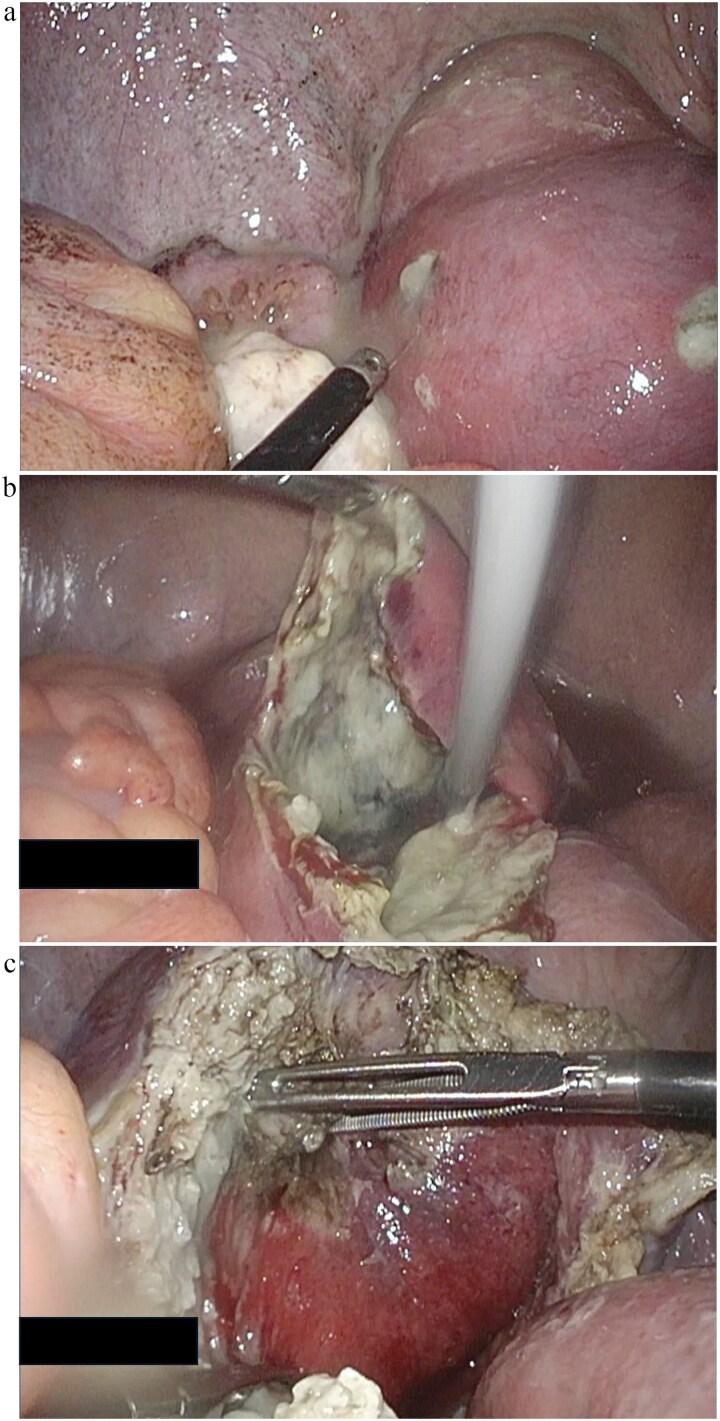
Laparoscopic surgical images of the case: (a) Two perforations in the uterine body, sites of purulent discharge. (b) Findings after incising the uterine muscle layer to connect the two perforation sites and draining the cavity. (c) Findings after partial resection of the uterine wall.

**Figure 3:**
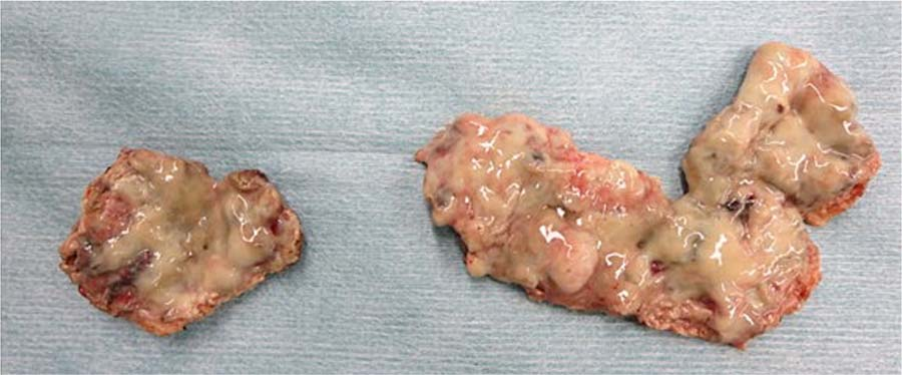
Resected uterine wall: An abscess adherent to the inner surface of the resected uterine wall.

Emergency laparoscopic surgery was initiated for diffuse peritonitis. Although copious purulent ascites was observed, no gastrointestinal perforation was identified. Instead, two perforations with purulent discharge were found in the enlarged uterine body, confirming ruptured pyometra (Fig. 2a). Given the patient’s advanced age, comorbidities, and hemodynamic instability, hysterectomy was considered excessively invasive. The uterine wall was therefore incised, and purulent material was drained (Fig. 2b). Partial resection of the uterine wall was performed to maintain an open abscess cavity and facilitate continuous drainage (Fig. 2c). The abdominal cavity was irrigated with 8 L of warm saline, and drains were placed in the bilateral subdiaphragmatic spaces, uterine cavity, and Douglas pouch. Operative time was 2 h 4 min with minimal blood loss. Histopathology showed inflammatory cell infiltration with edema and necrosis, without evidence of malignancy (Fig. 3).

### Postoperative course

**Figure 4:**
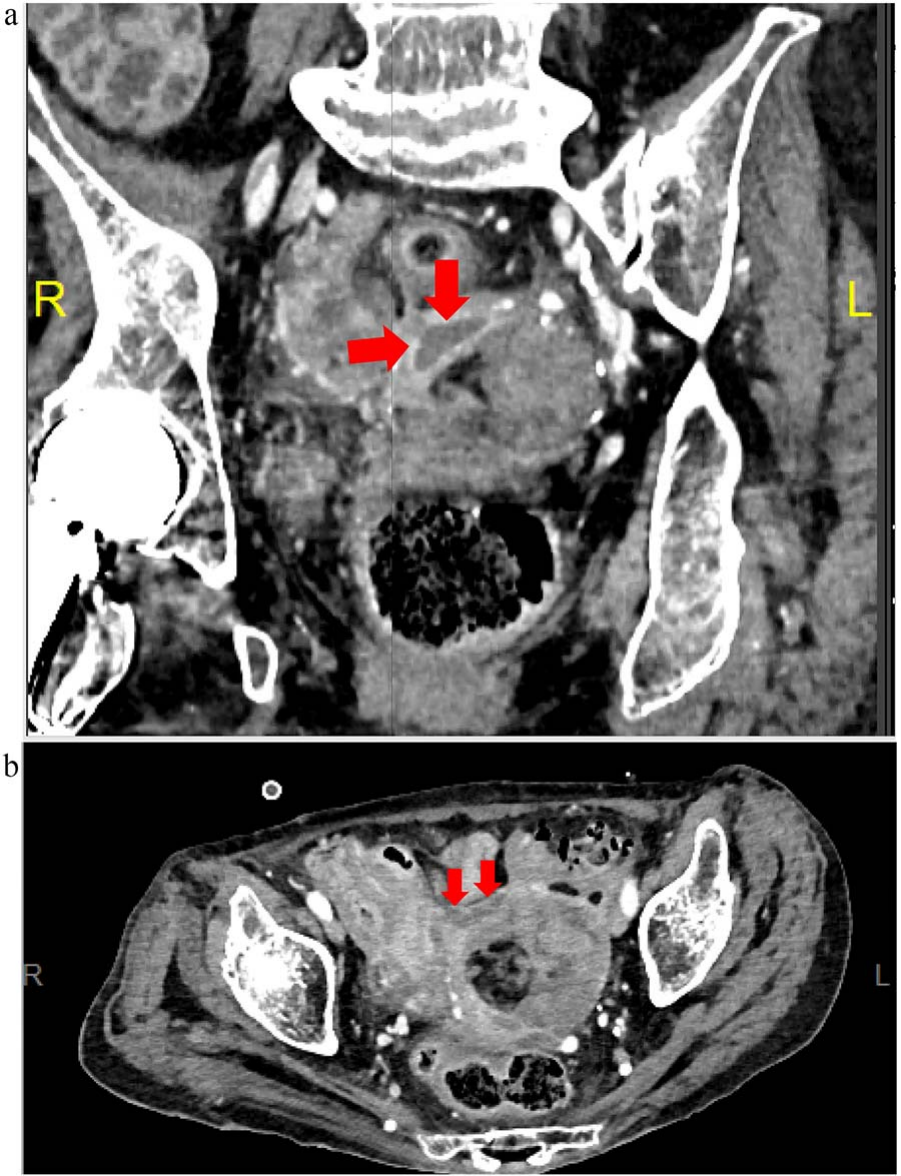
Postoperative Day 18 contrast-enhanced CT images (equilibrium phase). (a) Coronal view; (b) horizontal view.

Meropenem was administered postoperatively and later de-escalated to ampicillin/sulbactam based on culture results, which identified anaerobic organisms. Inflammatory markers gradually improved, and initial drains were removed. Although inflammation persisted mildly, follow-up CT on postoperative Days 3 and 10 showed no abscess formation. On postoperative Day 18, fever recurred, and CT revealed a pelvic abscess (Fig. 4). Transvaginal drainage using a Nelaton catheter aspirated 2.3 mL of purulent fluid, resulting in clinical improvement. Antibiotics were discontinued on postoperative Day 31. No additional surgery was required, and the patient was transferred to a long-term care facility.

## Discussion

Ruptured pyometra predominantly affects postmenopausal women and carries a high mortality risk when complicated by peritonitis or septic shock [1, 2, 5]. Although hysterectomy has traditionally been regarded as standard treatment, its invasiveness poses substantial risks in elderly or frail patients [3, 4]. In such cases, prognosis depends more on physiological tolerance than on definitive local disease control.

The primary goal in emergent management is rapid source control through effective drainage and thorough lavage [1, 2, 6]. Complete uterine removal is not always necessary, particularly when intraperitoneal contamination is already present [4, 7]. Drainage-based management should therefore be considered part of a staged strategy rather than a definitive alternative to hysterectomy [3, 7]. This concept is consistent with damage control principles widely applied in emergency surgery, in which abbreviated procedures are performed to stabilize critically ill patients before considering definitive surgery.

Malignancy is frequently associated with pyometra in elderly patients, with reported rates of 23%–35% [8–12]. Immediate hysterectomy without adequate evaluation may result in suboptimal oncological management. Drainage prioritizes survival and allows time for further assessment. In addition, advanced malignancy may render radical surgery technically difficult or oncologically insufficient, further supporting the rationale for an initial drainage-based approach in unstable patients.

In laparoscopic uterine-preserving surgery, closure of uterine perforation has been reported; however, this may recreate a closed infected space, particularly in patients with cervical stenosis. In the present case, the uterus itself constituted the primary source of infection; therefore, the uterine wall was intentionally incised and partially resected to widely open the abscess cavity and prevent re-closure. This allowed thorough irrigation of the uterine cavity and sustained drainage, consistent with fundamental infection control principles. Rather than anatomical repair, maintaining effective drainage was prioritized to achieve reliable source control in a life-threatening setting.

Postoperative recurrence remains a potential limitation of drainage-based management. In this case, a recurrent pelvic abscess developed postoperatively but was successfully managed with minimally invasive transvaginal drainage. This clinical course suggests that recurrence following open drainage does not necessarily indicate treatment failure, but may represent a manageable complication within a staged therapeutic strategy. As reports of uterine-preserving drainage in elderly or critically ill patients continue to accumulate, this approach may become an increasingly important option in selected high-risk cases.

## Conclusion

Laparoscopic open drainage without hysterectomy can be a life-saving option for ruptured pyometra in selected high-risk patients. Individualized surgical strategies prioritizing source control and systemic stabilization are essential, particularly in elderly patients with severe comorbidities.

### Learning points

- Ruptured pyometra is a surgical emergency in which rapid source control is more critical than definitive anatomical repair, especially in elderly or frail patients.
- In uterine-preserving laparoscopic surgery, closure of uterine perforation may recreate a closed infected space; open drainage can facilitate effective infection control.
- Laparoscopic open drainage represents a feasible and less invasive alternative to hysterectomy in selected high-risk patients with ruptured pyometra.
